# Intelligence model on sequence-based prediction of PPI using AISSO deep concept with hyperparameter tuning process

**DOI:** 10.1038/s41598-024-72558-x

**Published:** 2024-09-18

**Authors:** Preeti Thareja, Rajender Singh Chhillar, Sandeep Dalal, Sarita Simaiya, Umesh Kumar Lilhore, Roobaea Alroobaea, Majed Alsafyani, Abdullah M. Baqasah, Sultan Algarni

**Affiliations:** 1 DCSA, Maharshi Dayanand University, Rohtak, Haryana India; 2 Arba Minch University, Arba Minch, Ethiopia; 3 Department of Computer Science and Engineering, Galgotias University, Greater Noida, UP India; 4 Department of Computer Science, College of Computers and Information Technology, Taif University, P. O. Box 11099, 21944 Taif, Saudi Arabia; 5 Department of Information Systems, Faculty of Computing and Information Technology, King Abdulaziz University, 21589 Jeddah, Saudi Arabia; 6 Department of Information Technology, College of Computers and Information Technology, Taif University, P. O. Box 11099, Taif, 21944 Saudi Arabia

**Keywords:** PPI prediction, Sequence-dependent features, Gene ontology (GO), Improved recurrent neural network, Deep belief network, Aquilla influenced shark smell optimization (AISSO), Engineering, Biomedical engineering

## Abstract

Protein–protein interaction (PPI) prediction is vital for interpreting biological activities. Even though many diverse sorts of data and machine learning approaches have been employed in PPI prediction, performance still has to be enhanced. As a result, we adopted an Aquilla Influenced Shark Smell (AISSO)-based hybrid prediction technique to construct a sequence-dependent PPI prediction model. This model has two stages of operation: feature extraction and prediction. Along with sequence-based and Gene Ontology features, unique features were produced in the feature extraction stage utilizing the improved semantic similarity technique, which may deliver reliable findings. These collected characteristics were then sent to the prediction step, and hybrid neural networks, such as the Improved Recurrent Neural Network and Deep Belief Networks, were used to predict the PPI using modified score level fusion. These neural networks’ weight variables were adjusted utilizing a unique optimal methodology called Aquila Influenced Shark Smell (AISSO), and the outcomes showed that the developed model had attained an accuracy of around 88%, which is much better than the traditional methods; this model AISSO-based PPI prediction can provide precise and effective predictions.

## Introduction

Amino acids comprise the bio-molecules known as proteins that cells require to survive everyday tasks. They are essential in biology because they connect numerous significant bioactivities of cells to Protein–Protein interactions (PPIs)^1–3^, allowing for a range of biological functions, autophagy, and immune function. Despite advances in genomics, proteomics, and genome biology, the functionality of more excellent sequenced proteins remains uncertain. The research of the interaction of a recognized target protein with unidentified proteins aids in discovering unknown protein functioning. The structure of protein interactions proposes developing novel therapeutics by supplying biological routes present in the target’s surroundings^4^.

Numerous fields of neurology, cell biology, and developmental biology have shown the value of optical regulation of protein–protein interactions^5,6^. Drug targets must be precisely identified and defined during the research and development phase. The foundation of computational formulas and system modeling is analytical data^7,8^. The tandem affinity purification^9^ and proteomics chips^10^, including microarray technologies, have been employed to forecast PPIs from protein complexes. Unfortunately, as protein data accumulates, these approaches encounter time and expense constraints and cannot match the demands of human life scientific studies as in the post-genomic age. Also, it is profitable to mention that the presence of subjective or objective variables, including activity as well as experiment error, causes experimental findings to diverge significantly from the actual outcomes, occasionally resulting in a high fraction of false-positive and maybe even false-negative experimental findings^11–18^.

A common method for determining the most discriminative features for multi-class classification is linear discriminant analysis (LDA). Deep recognition models have performed remarkably over the last ten years^19,20^. Except for the yeast, wherein diverse characteristics have been extensively investigated, amino acid sequence-dependent predictors constitute a large proportion of the publications on computationally anticipating proteome-wide PPIs. Features derived from amino acid sequences and their physicochemical qualities are used in sequence-dependent predictors. Auto covariance (AC), conjoint triads (CT), and pseudo amino acid composition (PSEAAC) were examples of feature models that have frequently been employed for predicting PPIs^21,22^.

Traditional biophysical approaches for PPI detection are both time-consuming and costly. Conventional computational strategies, on the other hand, demand prior knowledge of genomic and phylogenetic schematics and sequence interpretation to produce acceptable PPI predictive performance^23^. Machine learning (ML) approaches, such as Artificial Neural Networks (ANN)^24^, Support Vector Machines (SVM)^25^, and deep learning^26,27^, provide critical means for prudent prognosis of PPIs premised on the straightforward derivation of protein data from amino acid sequences, demonstrating that deep-learning systems can manage huge raw as well as complicated information and effortlessly learn beneficial and much more conceptual features in the task of PPI prediction. As a result, we created an AISSO-based deep concept with a hyperparameter tuning approach for accurate and reliable PPI prediction. This work’s notable contributions have been listed below.

- We created an enhanced semantic similarity-based feature in the feature extraction process along with other features, which will aid in obtaining accurate findings.
- To provide an accurate forecast, an Improved RNN is developed to ensure the minimization of loss.
- A unique Aquila Influenced Shark Smell optimization is created to adjust the two classifiers’ weights, which shows efficient prediction.

The coordination of this article is as follows. Section “Literature survey” offers a synopsis of prior works on PPI prediction, Section “Problem statement” explains the problem statement of the research, Section “Proposed method” describes our suggested method for AISSO PPI prediction that is sequence-dependent, Section “Results and discussion” illustrates the results of the experiments, and Section “Conclusion” concludes the work, and the following section lists references.

## Literature survey

Some of the works related to PPI prediction were briefly reviewed in this section.

Patrick et al.^28^ created a computational strategy for developing precise protein complex structures. In this case, the AlphaFold2 is being used to forecast heterodimeric protein complexes. Models are created by using AlphaFold2 methodology and optimized multi-sequence alignment. A simple formula was built utilizing the projected interfaces to predict the DockQ score, separating satisfactory from wrong designs and associating non-interacting proteins with state-of-the-art accuracy. Even though this approach can yield excellent predictions, it only addresses protein complex structures in their heterodimeric form, even though every protein chain in such complexes might also have homodimer topologies or even other higher-order modes.

Satyajit et al.^29^ developed the AVPSO approach for PPI prediction, which is utilized to choose the optimum collection of features. The ideal feature subset gets utilized to forecast the PPIs by employing the light gradient boosting machine (LGBM) algorithm. This suggested model AVPSO-LGBM attained around 97% accuracy rate as well as around 95% in the fivefold CV assessment. The AV-PSO-LGBM beats conventional methodologies regarding prediction accuracy, indicating its generalization capabilities.

DeepTrio, a sequence-dependent strategy for predicting PPI utilizing mask multi-parallel CNNs, was reported by Xiaotian et al.^30^. DeepTrio offers improved PPI prediction and an understandable depiction of the significance of every protein sequence in both online and offline implementations. DeepTrio is being upgraded to give further perspectives on the influence of every input node on prediction outcomes.

Yang et al*.*^31^ established multiple modal protein pre-training paradigms with three modes: sequence, structural, as well as function (S2F). Interestingly, this approach encodes the structural characteristic using the topological complexity of heavy atom point clouds. It enables the system to gain structural data regarding the backbones and branched chains. Furthermore, this approach integrates information from the operational descriptions of proteins acquired from research or hand annotations. The experimental outcomes reveal that the S2F trains protein embeddings and works well on a multitude of PPI tasks.

Chiara et al*.*^32^ developed a revolutionary technique that was applied in a publicly accessible tool, “PepThreader,” to anticipate and analyze PPIs. PepThreader threads numerous segments produced from a full-length protein sequence over a secondary peptide template combined with a target protein, “spotting” promising linking peptides and rating it on a threading score (TS) that depends on structure and sequence. The TS process begins with a scoring system that depends on the sequence resembling peptides. Following that, the original hits are reranked utilizing structure-dependent scoring methods.

Bin et al*.*^33^ suggested a unique deep-forest-dependent strategy for predicting PPIs. PPI patterns are firstly retrieved and subsequently constructed in this work. Next, an elastic net is used to enhance the prediction effectiveness by optimizing the initial feature vectors. Ultimately, a deep forest-dependent GcForest-PPI model is constructed. The finding suggests that GcForest-PPI may increase prediction accuracy, aid in supplement studies, and aid drug development.

Li et al*.*^34^ suggested SDNN-PPI, a PPI forecasting methodology built on self as well as deep learning. To more precisely forecast PPIs, this tactic employs self-attention to optimize DNN feature retrieval. There was a fivefold CV to assess the generalization capabilities of SDNN-PPI. The one-core and crossover networks are used extensively to assess the model’s merits and drawbacks and forecast PPIs. The findings also revealed that the system appropriately forecasts the interaction pairings in the network.

Zeng et al*.*^35^ created Deep PPISP, a unique deep learning-dependent system for PPI site prediction that blends local contextual and global sequence information. A sliding window was utilized to collect characteristics of neighbours of a target amino acid for local contextual information. A text CNN model is being used to retrieve features from the entire protein sequence for global sequence characteristics. Then, the local contextual and global sequence information are integrated to anticipate PPI sites.

Wu et al.^36^ have deployed more insightful feature extraction made possible by this module’s effective capture of pertinent patterns and representations found in protein sequences. To ascertain the relationships between pairs of input proteins, the paper built a novel FRN that was incorporated into our model’s Global Feature Extraction module. The FRN efficiently captures the underlying relational information between proteins by enhancing PPI predictions. In sequence-based PPI prediction, the DL-PPI framework exhibits cutting-edge performance.

Valverde et al.^37^ have introduced a brand-new deep learning framework called DPPI that can be used to model and forecast PPIs using sequence data. Our model effectively uses evolutionary information of a protein pair under prediction as well as existing high-quality experimental PPI data, combining a deep, Siamese-like convolutional neural network with random projection and data augmentation to predict PPIs. According to our experimental data, DPPI performs more computationally efficiently and beats state-of-the-art approaches on some benchmarks regarding the area under the precision-recall curve.

Jha et al.^38^ have exploited the structural information and sequence properties of proteins; we apply a graph convolutional network (GCN) and graph attention network (GAT) to predict the interaction between proteins. We construct protein graphs using the PDB files, which include three-dimensional atomic coordinates. The protein graph represents the residue interaction network, sometimes called the amino acid network, in which every node is a residue. They are connected if two nodes contain two atoms (one from each node) inside the threshold distance. We employ the protein language model to extract the node/residue features. The protein sequence serves as the language model’s input, while the feature vectors for each amino acid in the underlying sequence serve as its output. Table 1 shows the reviews of conventional models.

**Table 1:** Reviews of conventional models.

Author	Deployed schemes	Features	Challenges
Patrick et al*.*^28^	AlphaFold2	Modelling mono-chain protein structures with incredible precision	The forecasted interacting partners have a significant impact on system performance
Satyajit et al*.*^29^	Light Gradient Boosting Machine (LGBM) with embedded feature selection	It is not affected by the classifiers	PSO has a decreased rate of convergence in the iterative process
Xiaotian et al*.*^30^	DeepTrio	Provide better performance in different datasets	Still need improvement in PPI critical region prediction
Yang et al*.*^31^	Sequence-structure–function (S2F) transformer model	As inputs, no structural or functional data is required for downstream PPI operations	Homology and structure-split Validation increase the system’s complexity
Chiara et al*.*^32^	PepThreader	Simple and less time-consuming	PepThreader cannot always distinguish the real binding peptide from a pool of candidate binders
Yu et al*.* ^33^	GcForest-PPI	It is suitable for cross-species prediction as well	The comprehensive critical characteristics of PPIs are yet to be known
Li et al*.*^34^	SDNN-PPI	Achieves high accuracy	Results may vary. To address these problems, an ensemble meta-learning technique can be created that is adaptable to various domains and dependent on the datasets
Zeng et al*.* ^35^	TextCNN	It uses local and global features for better prediction	Bad at forecasting long-length proteins

### Problem statement

A living thing’s necessary component is protein. Predicting PPIs significantly affects illness prevention, medicine development, and our comprehension of life’s behavioural processes. While the advancement of high-throughput technology allows for identifying PPIs in large-scale biological research, time, cost, false positive rate, and other constraints limit the extensive application of experimental approaches. To predict PPIs quickly and reliably, computational methods are therefore desperately needed as a supplement to experimental methods.

Class imbalance occurs when there are significantly fewer interacting protein pairs than non-interacting pairs in PPI datasets. Extracting meaningful information from protein sequences without overfitting or losing information is difficult. It is still difficult to interpret the predictions of sophisticated deep learning models, particularly in biological applications where interpretability is crucial for experimental validation and advancement. The model must function effectively on unknown proteins and interactions for practical use. Although many elevated experimental methods have been created to predict the PPIs, those have limitations like high cost and time consumption, and the selected features and classifiers are inappropriate and inefficient. However, the protein interaction found by experimental methods can only account for a small portion of the entire PPI networks because biological experiment methods are expensive and time-consuming. Furthermore, the detection results may have false positives and negatives due to the experimental setup and operational procedures. Thus, it is essential from a practical standpoint to create trustworthy computational techniques for reliably predicting protein interactions.

## Proposed method

Protein–protein interaction (PPI) prediction was important for understanding biological activities. Even though many different types of data and machine learning technologies have been employed in PPI prediction, effectiveness still has to be improved. Consequently, this paper presents a unique PPI prediction approach with two working phases: feature extraction and prediction. In the first phase, in addition to the traditional characteristics such as sequence-dependent and Gene ontology, we produced additional features using the semantic similarity approach, which would aid in accurate prediction.

**Fig. 1:**
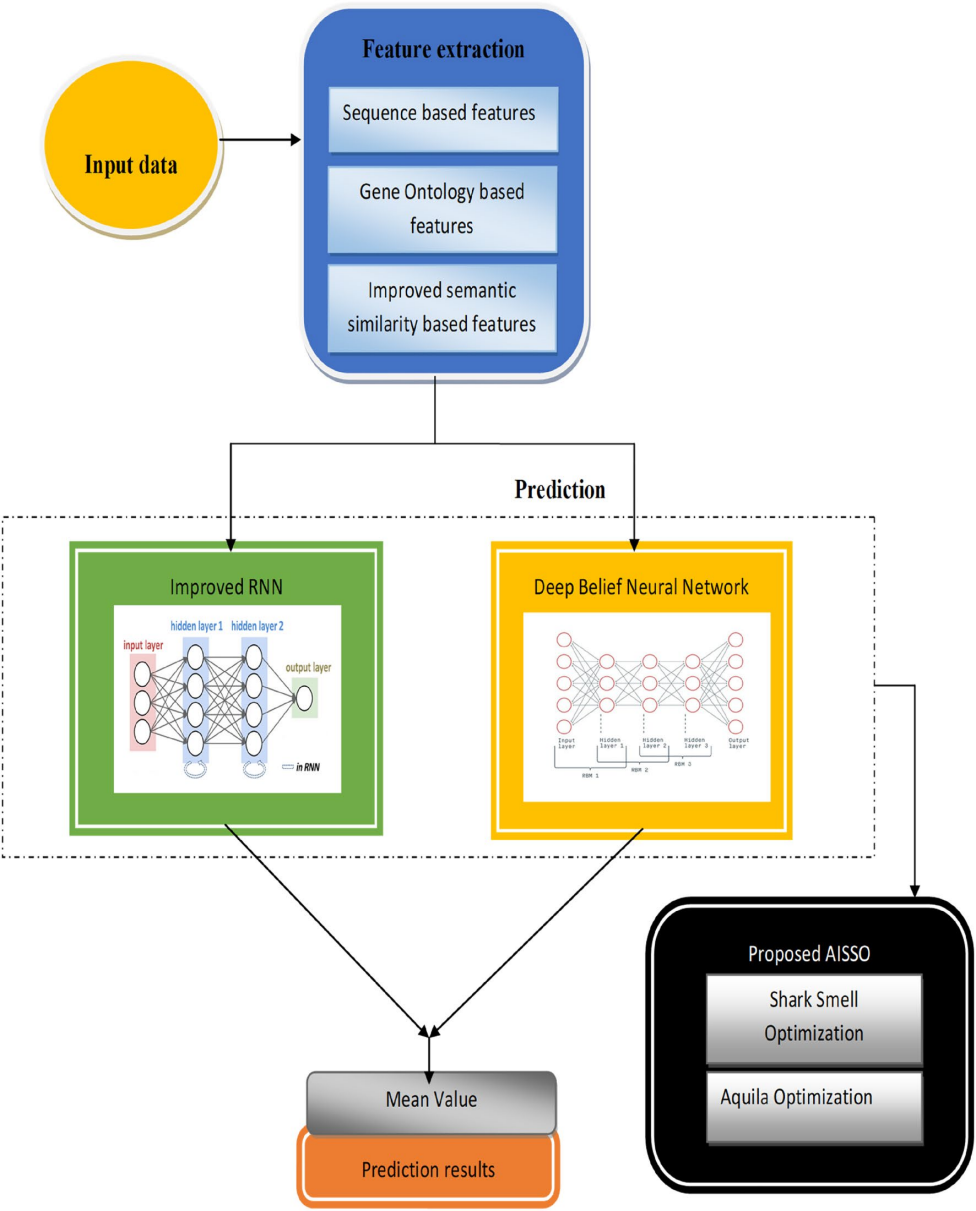
Architecture of the proposed AISSO-based PPI prediction model.

AISSO neural networks such as DBN and upgraded RNN were used in the second stage for better prediction. In addition, a unique optimization termed Aquila Influenced Shark Smell was developed in this work to provide a better and more reliable prediction by optimizing the weighting parameters. Figure 1 depicts the architectural design of the proposed PPI prediction approach, and a thorough description of our proposed work follows.

### Feature extraction

This work extracted three features from the given inputs: sequence-based physicochemical features, Gene Ontology (GO) based features, and semantic similarity-based features. A brief description of the feature extraction process is given below.

#### Sequence-based physicochemical features

The proteins have been utilizing twelve physical and chemical characteristics within its combined amino acids as the principle for PPI prognostication: hydrophilicity, adaptability, convenience, turn the scale, external surface, polarizability, antigenic tendency, hydrophobicity, net charge indicators of the side chains, polarity, solvent obtainable surface region, as well as side-chain volume. Hydrophobicity and polarity were the 12 qualities assessed on two distinct scales. The scores of the twenty critical amino acids’ physical–chemical property scales are listed in^35^. Every amino acid gets converted into a vector of 14 numerical data, one for each physicochemical scale rating. Because proteins fluctuate in length, they could be depicted by a varying count of vectors.

Alternatively, classification within an ensemble Meta-learning, including an ANN, k-NN, or NB, demands consistent feed. To generate a unified feed for the learner’s classification of the ensemble meta-base, the protein description is converted in a consistent vector form with auto-covariance (AC), whereby all proteins having different quantities of amino acids get portrayed by the identical length vectors. The AC of a protein sequence’s physicochemical characteristic scale describes the average correlations among amino acids split by a specific spacing over the complete protein sequence. This spacing between an amino acid and its neighbour is indicated here as a specific count of residues. The ${l}_{th}$ physical–chemical property scale’s AC for protein P, $A{C}_{l,g}$ is given by

1 $$A{C}_{l,g}=\frac{1}{L-g}{\sum }_{m=1}^{L-g}\left({P}_{l,m}-{\gamma }_{l}\right)\times \left({P}_{l,m+g}-{\gamma }_{l}\right)$$

2 $${\gamma }_{l}=\frac{1}{L}{\sum }_{m=1}^{L}{P}_{l,m}$$

where g is the preset gap, L is the P’s length,${\gamma }_{l}$ is the average of the ${l}_{th}$ physical–chemical scale values for *P*. By defining the maximum range to G $\left(i.e. g = 1, 2, 3,..., G\right)$, every protein may be initialized of $k\times GAC$ elements, where k seems to be the physicochemical property scales count.

Original physicochemical scale data is converted into a unified vectorial format utilizing AC between amino acids. Consequently, irrespective of length, every protein may be described by the same length vectors. Despite their varied lengths, proteins P1 and P2 were expressed by vectors of 28 AC values. To eliminate variance impacts, set the mean of every feature to zero standard deviation to one, as shown below:

3 $${S}_{l}=\frac{{\alpha }_{l}-{\gamma }_{l}}{S{D}_{l}},l=1,....M,$$

where ${S}_{l}$ denotes the normalized value, ${\alpha }_{l}$ represents the raw value of the ${l}_{th}$ AC, ${\gamma }_{l}$ and $S{D}_{l}$ represents the mean as well as the standard deviation of the ${l}_{th}$ AC, while M denotes the multitude of AC values inside the AC vector.

Furthermore, we have used a min–max scaling approach to scale the normalized AC values to a predetermined range of [0, 1] to guarantee that the ACs produced via diverse physical chemical scales were proportionate and will lessen the effect of outliers even more. Equation (4) describes the min–max scaling.

4 $$Scal{e}_{l}=\frac{{S}_{l}-MI{N}_{l}}{MA{X}_{l}-MI{N}_{l}},l=1,...M,$$

where $Scal{e}_{l}$ seems to be the scaled value, ${S}_{l}$ represents the ${l}_{th}$ AC’s standardized value,$MA{X}_{l}$ as well as $MI{N}_{l}$ were the maximum as well as a minimum of the ${l}_{th}$ AC’s standardized values, respectively.

#### Gene ontology (GO) feature extraction

GO seems to be a systematic vocabulary for identifying gene functionalities, including their links to molecular functioning, cellular elements, and biological processes. Each subontology would be expressed as a grounded DAG, in which Every link indicates a connection of two contexts (*part_of*, *is_a*), and every node correlates to a GO-term. This hierarchy helps understand operational interactions among genes and has been highly beneficial in appraising the significance of genes’ involvement in diverse biological processes, notably PPI prediction.

We have used a method that classifies protein pairings by clustering GO terms. We explore the GO hierarchy from the GO terms in ${G}_{u}$ as well as ${G}_{v}$ up to their lowest common ancestor (ULCA), two given sets of GO terms ${G}_{u}$, and ${G}_{v}$ tagging each of the proteins ${p}_{u}$ as well as ${p}_{v}$ in a pair. In this way, we may determine the LCA of every protein pairing $<{p}_{u},{p}_{v}>$ in a specified collection of protein pairings.

The identified LCAs are stored in a list sorted according to hierarchical GO levels. Except for those already assigned to a pre-existing cluster, each LCA was regularly aggregated in the set of sorted lists to create a cluster. Consequently, the entire GO-terms DAG is split into a set of mutually exclusive subgraphs anchored by an LCA.

GO-term feature vectors were created by assessing the existence or non-presence of common GO words or by assigning them a weight based on the local topology and the data they contain. Alternatively, one GO-dependent feature is defined as a GO group referenced by LCA. To convert these annotated groups ${G}_{u}$ and ${G}_{v}$ for every protein pairing $<{p}_{u},{p}_{v}>$ into GO-based numerical values that LCA indexes, initially find the GO terms in sets ${G}_{u}$ as well as ${G}_{v}$ on every LCA-indexed subgraph. We calculate the nodes along the rising route up to the base of a subgraph for every GO term and add the node numbers on the subgraph. The value of the matching GO term feature gets allocated to this sum.

#### Improved semantic similarity based feature extraction

When annotations were plain texts, syntactic similarity alone cannot determine the proximity between sources. Tags generally struggle with heterogeneity as well as ambiguous issues, in which taggers may use multiple words with identical meanings or even the same word with distinct meanings. As a result, while comparing and identifying resemblance, SSD retrieves semantic relations. The degree of similarity has been calculated using the Semantic Similarity Identification approach. Each source is mapped to compute the similarity. Specifically, the vector model suffers from problems including missing semantic data and word impropriety (e.g., ignore synonymy). The PWR approach eliminates vector semantic issues by integrating symbolic features into the matrix form. The SSD approach’s main focus in this application is to use a cosine similarity measure to identify the relationships between each pair of resources. SSD cosine similarity incorporates both syntactic and semantic similarity.

5 $$Se{m}_{sim}\left({R}_{a},{R}_{b}\right)=\frac{{R}_{b}.{R}_{a}}{\left|{R}_{b}\right|.\left|{R}_{a}\right|}=\frac{{\sum }_{a=1}^{m}\left({\omega }_{b}*SR.{\omega }_{a}\right)}{\sqrt{{\sum }_{a=1}^{m}{\left({\omega }_{b}^{a}*SR\right)}^{2}}.{\sum }_{a=1}^{m}{\omega }_{a}^{2}}$$

In this work, the improved semantic similarity is used to know the relation between two GO terms $\left({R}_{a},{R}_{b}\right)$.

6 $$Se{m}_{sim}\left({R}_{a},{R}_{b}\right)=\frac{{\sum }_{a=1}^{m}\sqrt{{R}_{a}{R}_{b}}*{\omega }_{a}}{\sqrt{{\sum }_{a=1}^{m}\left({R}_{a}\right)}\sqrt{{\sum }_{a=1}^{m}\left({R}_{a}\right)}}$$

${\omega }_{a}$ is the weight generated for each protein sequence. The weight is calculated using the cubic map function.

7 $${\rm E}_{c+1}=\rho {E}_{c}\left(1-{E}_{c}^{2}\right){E}_{c}\in \left(\text{0,1}\right)$$

This improved semantic similarity method was utilized in this work to obtain the best and most appropriate features.

### Prediction phase

**Fig. 2:**
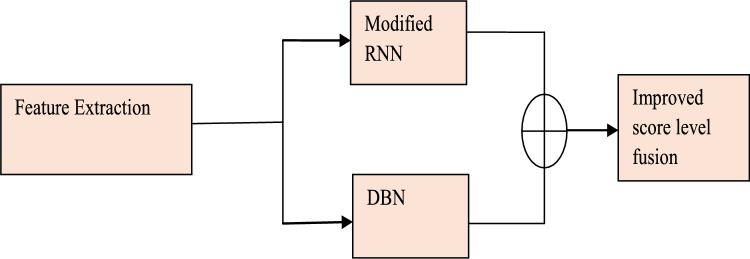
Prediction phase model.

The prediction model applies the retrieved characteristics and uses a hybrid model incorporating the classifiers from Deep Belief Networks and Improved Recurrent Neural Networks. Figure 2 shows the prediction phase model. The idea behind the hybrid is as follows: The characteristics are first passed to each of the two classifiers individually, and the final result is determined by averaging the output of the classifiers using modified score-level fusion. Here, the suggested AISSO is used to train both classifiers by adjusting the ideal weights, improving the prediction outputs’ performance.

#### Improved recurrent neural network (RNN)

RNNs are a kind of neural net wherein the links between functional blocks create a circle. Except for feed-forward networks, RNNs may handle arbitrary sequences of inputs utilizing their internal memory. An RNN’s computational units each have a time-dependent actual valued activation and a configurable weight. RNNs were produced by iteratively integrating the very same collection of weights across a graph-like structure. Many RNNs use Eq. (8) to specify the values of their concealed blocks.

8 $${H}^{t}=F\left({H}^{t-1},{x}^{t};\vartheta \right)$$

The learned architecture still has an identical input size because RNN has been defined from the perspective of migration from one stage to another. Furthermore, the design utilizes a unique transitional formula having identical attributes for every time interval. Long Short-Term Memory (LSTM) is another RNN in which LSTM cells substitute the classic hidden layers. Those cells were composed of multiple gates that could govern the input stream. An LSTM cell has four gates: input gateway, cell state, forget gate, and output gate. This has a sigmoid tier, a tanh layer, and point-wise multiplication. The following were the multiple gates as well as their operations:

- Input gate: This input gate is generally comprised of inputs. Some retrieved characteristics would be utilized as input in our work.
- Cell State: The system runs throughout and has gates allowing it to add and remove data.
- Forget gate: Specifies the level of knowledge that’ll be permitted.
- Output gate: LSTM’s output makes up this component.
- Integers from 0 to 1 are output by the sigmoid layer, indicating how much of each component can move.
- A new vector created by the Tanh layer is added to the state.

The cell status gets modified depending on the gate output. The accompanying formulas have been used to express it mathematically.

9 $${F}_{t}=\theta \left({W}_{F}.\left[{H}_{t-1},{x}_{t}\right]+{d}_{F}\right)$$

10 $${i}_{t}=\theta \left({W}_{i}.\left[{H}_{t-1},{x}_{t}\right]+{d}_{i}\right)$$

11 $${e}_{t}=\mathit{tan}h\left({W}_{e}.\left[{H}_{t-1},{x}_{t}\right]+{d}_{e}\right)$$

12 $${q}_{t}=\theta \left({W}_{q}\left[{H}_{t-1},{x}_{t}\right]+{b}_{q}\right)$$

13 $${H}_{t}={q}_{t}*\mathit{tan}h\left({e}_{t}\right)$$

where ${x}_{t}$ seems to be the input vector,${H}_{t}$ indicates the vector of output, ${e}_{t}$ would be the cell state vector, ${F}_{t}$ is the vector for the forget gate, ${i}_{t}$ is the vector for the input gate, ${q}_{t}$ has been the vector for the output gate, while W, d has been the parameter weight matrix and vector. The tanh activation function, which is represented in this paper, is

14 $${H}_{t}=\mathit{tan}h\left({W}_{hh}{H}_{t-1}+{W}_{xh}{x}_{t}\right)$$

${W}_{hh}$ denotes the recurrent neuron weight and ${{\varvec{W}}}_{{\varvec{x}}{\varvec{h}}}$ denotes the input neuron weight.

The loss function measures the difference between an algorithm’s current and predicted output, assessing data mimicry. Cross-entropy is commonly used in machine learning for more robust generalization models and faster training. With binary and multiclass categorization issues, cross-entropy could be applied.

A binary regression model may be utilized to categorize observations into two groups. Particularly a vector of input characteristics x, the model’s output for a provided observation may be read as a probability that offers the foundation for categorizing the observation. The logistic function $Q(\varepsilon )=\frac{1}{\left(1+{e}^{-\varepsilon }\right)}$ is being used to describe the likelihood in a logistic regression, wherein z represents a function of the input vector $\varepsilon$, most frequently a linear function. Throughout most instances, logistic regression improves the log loss for all of the findings on which it is trained, which is identical to maximizing the sample’s average cross-entropy. For example, suppose we have N samples with each sample indexed by n = 1, 2…N. The *average* of the loss function is then given by

15 $$J(w)=\frac{1}{N}{\sum }_{n=1}^{N}C\left({\zeta }_{n},{\lambda }_{n}\right)=-\frac{1}{N}{\sum }_{n=1}^{N}\left[{z}_{n}{\mathit{log}\hat{z}}_{n}+\left(1-{z}_{n}\right)\mathit{log}\left(1-{\hat{z}}_{n}\right)\right],$$

where $${\hat{z}}_{n}=h\left(w.{\chi }_{n}\right)=\frac{1}{\left(1+{e}^{-w.{\chi }_{n}}\right)}$$

Cross-entropy loss is another name for logistic loss. It is sometimes referred to as log loss. This work uses the cross-entropy loss function below to lessen the model’s loss.

16 $$cros{s}_{Ent}=\frac{-1}{N}\left[{\sum }_{i=1}^{N}\left[{t}_{i}\mathit{log}\left(sigmoid\left(\chi \right)\right)+\left(1-{t}_{i}\right)\mathit{log}\left(sigmoid\left(1-{\varsigma }_{i}\right)\right)\right]\right]$$

#### Deep belief network

DBNs appear to be inventive techniques. A DBN is composed of stacked RBMs that engage in greedy application training to achieve good performance in an unsupervised environment. Training took place layer-by-layer in a DBN, executing each layer as an RBM trained on top of the previous layer. As a feed-forward network that allows for weight fine-tuning using an alternative strategy, DBNs are a group of RBM layers used for pre-training.

Since RBMs and auto-encoders can be pre-trained on unclassified data and fine-tuned on a small quantity of labelled data, their significant utilization is probably due to the lack of labelled data. A DBN was trained layer by layer using a greedy application training technique. It was used because the greedy application method optimizes each layer at a time in a greedy manner. A joint supervised training algorithm is typically applied to each layer during the fine-tuning stage that follows unsupervised training.

A greedy tier unsupervised approach was used in the pre-training phase to train the basic features, and a softmax layer was added to the top layer during the fine-tuning phase to improve the characteristics of the labelled samples. As shown in Eq. (17), the SD was normalised to graphically depict the complexity.

17 $${\delta }^{*}=\frac{\delta -{\delta }_{min}}{{\delta min}_{max}}$$

In this, v is each visible unit of RBM, while h is each hidden unit. The model’s three metrics were found to select the strategy.:$\phi =\left\{U,\Phi ,D\right\}$. The weight matrix U, the hidden layer component bias $\Phi$, as well as the visible layer component bias D.

Consider an RBM comprises of p hidden cells as well as q visible cells, with ${\upsilon }_{r}$ representing the *r*th visible unit &${\hslash }_{r}$ representing the *j*th hidden unit, with the attributes stated in Eq. (18):

18 $$\lambda =\left\{{\mu }_{r,j}\in {\xi }^{p\times q}\right\}$$

Here ${\mu }_{r,j}$ denotes the weighted average of the *r*th exposed cells, as well as the *j*th concealed cell from Eq. (19).

19 $$Z=\left\{{A}_{r}\in {\xi }^{m}\right\}$$

Here ${A}_{r}$ denotes the *r*th visible cell’s bias limit from Eq. (20);

20 $$Z=\left\{{C}_{j}\in {\xi }^{n}\right\}$$

where ${C}_{j}$ represents the *j*th visible cell’s bias threshold. The RBM energy formula has been expressed in Eq. (21) for (v, h) via the current state, assuming concealed as well as visible layers replicate the Bernoulli distribution.

21 $$E\left(y,h\left|\phi \right.\right)=-{\sum }_{r=1}^{n}{A}_{r}{v}_{r}-{\sum }_{j=1}^{m}{B}_{j}{h}_{j}-{\sum }_{r=1}^{n}{\sum }_{j=1}^{m}{v}_{r}{V}_{rj}{h}_{j}$$

$\phi =\left\{{V}_{rj},{A}_{r},{B}_{j}\right\}$ represented the attributes of the RBM prototype, and the operation of energy displayed the value of energy amongst estimations from every viewable node as well as every concealed layer node. Because of the energy function’s extension and expansion, the combined probability distribution formula was obtained, wherein the nodes collection of viewable layers as well as hidden layers nodes was in a particular state independently (v, h), as shown in formula (22):

22 $$P\left(v,h\left|\phi \right.\right)=\frac{{e}^{-E\left(v,h\left|\phi \right.\right)}}{Z\left(\phi \right)}$$

23 $$Z\left(\phi \right)={\sum }_{v,h}{e}^{-E\left(v,h\left|\phi \right.\right)}$$

where $Z\left(\phi \right)$ the normalized aspect or distributed function displays the overall energy estimates of all available states for the set of hidden nodes and visible layers in the expression (23). The parameters are often obtained by determining the probability function. After presenting the associated likelihood distribution $P\left(v,h\left|\phi \right.\right)$ the margin distributions $P\left(v\left|\phi \right.\right)$ of the viewable layer node collection might be obtained by adding the overall restrictions of the concealed layer node collection in Eq. (24):

24 $$P\left(v\left|\phi \right.\right)=\frac{1}{Z\left(\phi \right)}{\sum }_{h}{e}^{-E\left(v,h\left|\phi \right.\right)}$$

The marginal distributions represent the likelihood that the node configuration inside the visible layers fell within the designated level distribution. The RBM system’s extraordinary layer-layer connections and inter-layer connectionless form give it the following noteworthy requirements: The enactment conditions of every hidden layer cell were restrictively autonomous after the presentation of the visible cell circumstances. In this instance, the hidden component’s activation probability was as indicated by Eq. (25):

25 $$AP\left({h}_{j}=1\left|\phi \right.\right)=\sigma \left({B}_{j}+{\sum }_{i}{v}_{i}{V}_{ij}\right)$$

Consequently, upon stating the hidden components’ criterion, the visible components’ initiation probability likewise became uncorrelated, as seen by Eq. (26):

26 $$AP\left({v}_{r}=1\left|h\right.\right)=\sigma \left({A}_{r}+{\sum }_{j}{V}_{rj}{h}_{j}\right)$$

This was necessary to figure out the 3 model parameters before selecting the RBM model:$\phi =\left\{{V}_{ij},{A}_{r},{B}_{j}\right\}$ The logarithmic probability measures were used in the parametric organization to determine the parameters’ subordinates. According to Eq. (24), $P\left(v\left|\phi \right.\right)=\frac{1}{Z\left(\phi \right)}{\sum }_{h}{e}^{-E\left(v,h\left|\phi \right.\right)}$, Since energy E was determined by extending *P*, it would be inversely related to likelihood P. The inclination increase strategy, related to parameter adjustment as shown by Eq. (27), was the standard method for increasing functional probability.

27 $$\phi =\phi +\mu \frac{\partial \mathit{ln}P(v)}{\partial \phi }$$

This repetitive approach increased the probability P while decreasing the energy E. Table 2 shows the hyperparameters of classifiers.

**Table 2:** Classifier Hyper-parameters.

Classifier	Parameter
DBN	learning rate:0.01 Max Iter = 50.0; Batch Size = 200; Step Ratio = 0.01; Drop Out Rate = 0.1; Verbose = ’true’; hidden neuron: 50 Initial Momentum = 0.5; % momentum for first five iterations Final Momentum = 0.9; % momentum for remaining iterations Weight Cost = 0.0002; % costs of weight update Initial Momentum Iter = 5; Max Iter = 100; Step Ratio = 0.01; BatchSize = 0; Verbose = false;
RNN	input layer-1 lstm layer-1 fullyConnectedLayer-3 activation: tanh ‘optimizer’: ‘sgdm’,… ‘MaxEpochs’,50, … ‘MiniBatchSize’,70, … ‘Verbose’, false

#### Modified score level fusion

In score-level fusion, an individual’s identity is determined by consolidating the match scores produced by various biometric matches. Usually, the biometric system uses the single scalar score produced due to this consolidation process. The conventional score level fusion is given below. The conventional score level fusion has some limitations. Individual deep-learning models’ scores can be susceptible to noise or errors during prediction. These errors can propagate and affect the final fused score, potentially leading to misclassification. To overcome this, we have modified a new method for fusing scores of both deep learning prediction scores. To conventional score level fusion is given in Eq. (28),

28 $$F_{S} = \frac{{mRNN_{predictedscore} + DBN_{\Pr edictedscore} }}{2}$$

(i) The map of mRNN output prediction is provided in Eq. (29)
  29 $$S_{mRNN - Mape} = 100*\frac{1}{{N_{MRNN} }}\sum\limits_{i = 1}^{{N_{mRNN} }} {\frac{{y_{i} - S_{mRNN} }}{{y_{i} }}}$$
(ii) Also, compute Mape for DBN output is given in Eq. (30).
  $y_{i}$ is the actual score, $S_{mRNN}$ is the predicted score of modified RNN
  30 $$S_{mRNN - Mape} = 100*\frac{1}{{N_{DBN} }}\sum\limits_{i = 1}^{{N_{DBN} }} {\frac{{y_{i} - S_{DBN} }}{{y_{i} }}}$$
(iii) To fuse the score by using Eq. (31),
  31 $$FS = w_{1} *S_{mRNN} + w_{2} *S_{DBN}$$
  where $w_{1} ,w_{2}$ are the weights, these are provided in Eqs. (32) and (33). The weight can be calculated by using the above map values.

If $$S_{mRNN - Mape} \le S_{DBN - Mape}$$:

32 $$w_{1} = \frac{{S_{mRNN - Mape} }}{{S_{DBN - Mape} + S_{mRNN - Mape} }}$$

33 $$w_{2} = \frac{{S_{DBN - Mape} }}{{S_{DBN - Mape} + S_{mRNN - Mape} }}$$

This improved score level fusion technique was used to identify which deep model achieves a better prediction rate than others. Based on the minimum mape weight-based fusion technique to fuse the score accurately. This modified fusion technique can be tailored for improved performance.

### AISSO-based optimal training of hybrid classifier

The hunting techniques of eagles, particularly those of the Aquila genus, inspire AOA. Nature-inspired algorithms frequently present creative answers to optimization issues by imitating natural processes. AOA usually strikes a good balance between exploration and exploitation. Analogously to eagles hunting for prey, it uses processes to efficiently explore the search space and capitalize on potential places for better answers. Applications of AOA can be found in many different disciplines, including engineering, finance, logistics, and more, for a wide range of optimization problems. It is appropriate for a wide range of real-world applications due to its versatility. Good convergence properties are frequently exhibited by AOA, which means it can quickly and effectively converge to almost ideal solutions.

For applications that require speed, this efficiency is essential. To attain optimal performance, SSO, like many other optimization algorithms, must have its parameters adjusted. Choosing the right parameter values can be difficult and might require much experimentation. Despite its exploration–exploitation balance, SSO may nevertheless experience the problem of convergent to local optima, particularly in multimodal or highly nonlinear optimization environments. Developing escape strategies from local optima is crucial to enhancing its robustness.

Even though SSO draws inspiration from natural occurrences, its theoretical underpinnings might not be as solid as those of certain more known optimization techniques. Due to this lack of theoretical rigour, its behaviour under certain situations may be more difficult to evaluate and comprehend. The computational needs of SSO may become exorbitant depending on the size and complexity of the task. This could be a disadvantage, especially for real-time applications or large-scale optimization projects requiring much processing power. The combination of SSO and Aquila optimization is the proposed AISSO. SSO algorithm influences the Aquila update. The hybrid optimization idea outperforms the separate algorithms regarding speed and convergence rate.

#### Objective function and solution encoding

**Fig. 3:**
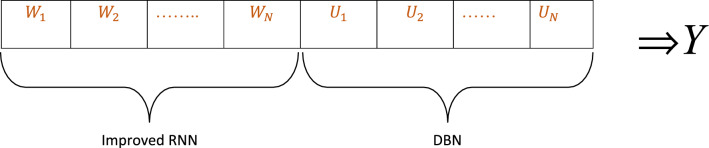
Proposed AISSO-based PPI prediction technique’s solution encoding.

The solution provided as input to the proposed AISSO-based model is shown in the following Fig. 3.

This work aims to minimize mean square errors, as indicated by Eq. (34). Initially, the attributes are provided to each of the two classifiers separately. The outcome is obtained by averaging the classifiers’ outputs using modified score-level fusion.

34 $$Obj=Min(MSE)$$

Here, the MSE is the mean square error.

The shark smell technique was motivated by the shark’s capability to hunt using its keen sense of smell. Several assumptions are taken into account when building the mathematical formulation^39^. They are as follows:

1. A fish gets hurt, so blood is injected into the water (the search space). Consequently, when contrasted to the shark’s motion velocity, the wounded fish’s velocity may be ignored; in another way, the source (prey) is considered to be set.
2. Blood is injected into the sea in a usual manner. The impact of water movement on odour-distorting particles is overlooked. The odour particles were more significant around the damaged fish. As a result, tracking the odour particles aids the shark in approaching its meal.
3. One damaged fish in the shark’s search area resulted in one odour source.

#### SSO initialization: first odour particles identification

When the shark detects an odour, the search activity starts. In reality, odour molecules from a wounded fish get poor diffusion (prey). A population of starting solutions for an optimization challenge in the viable search space is typically produced randomly to mimic this procedure. Each one of these options indicates an odour particle that indicates the shark’s likely location at the start of the search^40,41^.

35 $$\left[{Y}_{1}^{1},{Y}_{2}^{1},...{Y}_{NP}^{1}\right],$$

Where ${Y}_{1}^{1}$ denotes the population vector’s *y*th beginning location or ${y}^{th}$ the initial solution, while NP is the size of the population. The associated optimization issue is as follows:

36 $${Y}_{y}^{1}=\left[{Y}_{y,1}^{1},{Y}_{y,2}^{1},...,{Y}_{y,ND}^{1}\right]y=\text{1,2},...,ND,$$

ND = count of selection factors in the optimization issue, where ${Y}_{y,\tau }^{1}$= ${\tau }^{th}$ dimension of the shark’s *y*th position or $\tau \text{th}$ selection variable of the shark’s *y*th location ${Y}_{y}^{1}$.The strength of the odour at every location indicates its proximity to the prey. This SSO methodology uses an objective function to simulate this activity. A more significant objective function value signifies a more extraordinary odour, considering a maximization issue and using the basic rule. As a result, the shark is closer to its prey because of this procedure. As per this viewpoint, the SSO algorithm starts.

#### Shark movement toward the prey

At each point, the shark accelerates to get nearer to the prey. The baseline velocity vector may be represented as follows using position vectors:

37 $$V{V}_{1}^{1},V{V}_{2}^{1}...,V{V}_{NP}^{1}$$

The velocity vectors in Eq. (38) contain elements within every dimension.

38 $$V{V}_{y}^{1}=\left[V{V}_{y,1}^{1},V{V}_{y,2}^{1},....V{V}_{y,ND}^{1}\right]y=1,...,ND$$

The shark tracks the odour, and the strength of the odour dictates its motion; because of the higher intensity of the odour, the shark’s speed increases. This motion is quantitatively characterized by an objective function’s gradient from the optimisation standpoint. This gradient denotes the route wherein the function grows the fastest. This mechanism is depicted in Eq. (39).

39 $$V{V}_{y}^{{\ell}}={\eta }_{{\ell}}\,\cdot\,Rand1\,\cdot\,\nabla \left(OF\right){|}_{{x}_{i}^{{\ell}}}y=1,...,NP{\ell}=1,...,{{\ell}}_{max}$$

where $V{V}_{y}^{{\ell}}$= the shark’s approximate constant velocity; OF denotes the objective function; r = the objective function’s gradient; ${{\ell}}_{max}$ symbolizes the shark’s maximum count of phases for forward motion; ${\ell}$ = the count of steps, while Rand1 = a random value in the range [0,1]. Since a shark may not be able to attain the velocity predicted by the gradient function,${\eta }_{{\ell}}$ seems to be in the range [0,1]. The SSO algorithm’s Rand1 parameter allows for a more random search. The gravitational search method inspired the concept of considering Rand1 (GSA). Equation may be used to compute velocity in each dimension (40).

40 $$V{V}_{i,j}^{k}={\eta }_{{\ell}}\,\cdot\,Rand1\,\cdot\,{\left.\frac{\partial \left(OF\right)}{\partial {x}_{j}}\right|}_{{x}_{i,j}^{k}}y=1,...NP\tau =1,...,ND{\ell}=1,...{{\ell}}_{max}$$

Because of inertia, the shark’s velocity gets restricted, which is determined by its prior velocity. A simplified Eq. (41) is used to represent this process:

41 $$V{V}_{y,\tau }^{{\ell}}={\eta }_{{\ell}}\,\cdot\,Rand1\,\cdot\,{\left.\frac{\partial \left(OF\right)}{\partial {x}_{j}}\right|}_{{x}_{i,j}^{k}}+{\varphi }_{{\ell}}\,\cdot\,Rand2\,\cdot\,V{V}_{y,\tau }^{{\ell}-1}$$

$$y=1,...NP\tau =1,...,ND{\ell}=1,...,{{\ell}}_{max}$$

where ${\varphi }_{{\ell}}$ would be the momentum or inertia coefficient rate, which will have a value inside the interval [0,1] and become a constant for phase ℓ; Rand2 represents the momentum term’s randomized value source with a uniform dispersion on the interval [0,1]. The higher the amount of ${\varphi }_{{\ell}}$ The more inertia there is, the more dependent the present velocity is on the prior velocity. Using momentum contributes to clearer search pathways in the solution area from an arithmetical standpoint. The exploration in the algorithm becomes more diverse with Rand 2.

It is feasible to ignore or assign a very modest quantity to the shark’s baseline velocity before commencing the search strategy $V{V}_{y,\tau }^{0}$ for the velocity during the first phase $V{V}_{y,\tau }^{1}$. The shark’s speed may be enhanced up to a certain point. Unlike other fish, sharks do not have swim bladders to keep them afloat. As a result, they cannot stay still and must swim upward in a direction, even at a slow pace. This is accomplished by utilizing the powerful tail fin as a propulsion device. A shark’s typical speed is around 20 km/h, but it may reach 80 km/h while preparing to strike. The sharks’ peak to minimum velocities ratio is restricted. Equation (42) describes the velocity limiter utilized within every phase of the SSO method^8,42–44^.

42 $$\left|V{V}_{y,\tau }^{{\ell}}\right|=\mathit{min}\left[\left|{\eta }_{{\ell}}.{\left.Rand1.\frac{\partial \left(OF\right)}{\partial {x}_{\tau }}\right|}_{{x}_{y,\tau }^{{\ell}}}+{\varphi }_{{\ell}}.Rand2.V{V}_{y,\tau }^{{\ell}-1}\right|,\left|{\beta }_{{\ell}}.V{V}_{y,\tau }^{{\ell}-1}\right|\right],$$

$$y=1,...,NP,\tau =1,...,ND,{\ell}=1,...{{\ell}}_{max}$$

where ${\beta }_{{\ell}}$ is the level k velocity constraint ratio. Equation (42) calculates the value of $V{V}_{r,\tau }^{{\ell}}$ that has the identical symbol as the phrase chosen by the minimal operator in Eq. (42). Owing to the shark’s forward motion, its updated position ${I}_{y}^{{\ell}+1}$ was calculated using its prior velocity as well as position.

43a $${I}_{y}^{{\ell}+1}={x}_{y}^{{\ell}}+V{V}_{y}^{{\ell}}.\Delta {t}_{{\ell}}y=1,...NP{\ell}=1,...,{{\ell}}_{max}$$

where $\Delta {t}_{{\ell}}$ is the phase ${\ell}$ time interval. For the sake of convenience, $\Delta {t}_{{\ell}}$ is considered to be the same for all phases. Equation (42) yields every element of $V{V}_{y,\tau }^{{\ell}}\left(\tau =1,....,ND\right)$ of vector $V{V}_{y}^{{\ell}}$.

As the proposed logic, we use the Aquila Optimization’s updation function instead of SSO’s updation function. The random variables r1 and r2 are created using the random function, while the random variable r3 is generated using the ICMIC map.

43b $${x}_{{\ell}+1}=sm\left(\frac{\varpi }{{x}_{k}}\right)\varpi \in \left(0,\kappa \right){x}_{{\ell}}\in \left(-\text{1,1}\right)$$

When a high soar locates the prey location, the Aquila orbits its prey, positions itself, and then attacks. Contour flying with short glide aSSOult is the name given to this technique. In preparation for such an aSSOult, AO closely investigates the specified region of the intended prey. This behaviour is described numerically as Eq. (44).

44 $${X}_{2}\left(t+1\right)={X}_{best}(t)\times Levy({D}_{A})+{X}_{R}(t)+(\Pi -K)*ra,$$

where ${X}_{2}\left(t+1\right)$ is the outcome of the 2nd search method’s subsequent recapitulation of t. The dimension area is D, as well as the levy flight dispersion function is $Levy({D}_{A})$, that is the derivative of Eq. (44). At the *f*th iteration,${X}_{R}(t)$ is a randomized solution picked in the range of [1 N].

45 $$Levy({D}_{A})|=s\times \frac{R{I}_{1}\times \sigma }{{\left|R{I}_{2}\right|}^{\frac{1}{\beta }}}$$

where s represents a fixed value of 0.01, $R{I}_{1}$ and $R{I}_{2}$ represents a random integer among 0 & 1, $\sigma$ would be computed with the help of Eq. (45).

46 $$\sigma =\left(\frac{\psi \left(1+\beta \right)\times \mathit{sin}e\left(\frac{\pi \beta }{2}\right)}{\psi \left(\frac{1+\beta }{2}\right)\times \beta \times {2}^{\left(\frac{\beta -1}{2}\right)}}\right)$$

where $\beta$ is a fixed to 1.5. In Eq. (44), The values of y and x, which have been ascertained as follows, indicate the spiral pattern in the search.

47 $$\Pi =r\times \mathit{cos}(\vartheta )$$

48 $$K=r\times \mathit{sin}(\vartheta )$$

where

49 $$r={r}_{1}+\Upsilon \times {D}_{1}$$

50 $$\vartheta =-\omega \times {D}_{1}+{\vartheta }_{1}$$

51 $${\vartheta }_{1}=\frac{3\times \pi }{2}$$

$\Upsilon$ seems to be a small number with a value of 0.00565. ${D}_{A1}$ is an integer array that starts at one and moves to the search space length (Dim), and $\omega$ will be 0.005. The best shark positions were then chosen based on the greatest OF value.

In this study, Gauss mutation is used to provide a precise and trustworthy optimization. Gaussian mutation works by simply adding a random value from a Gaussian distribution to each vector member of an individual to create a new generation. Table 3 shows the optimization parameters.

**Table 3:** Optimization parameters.

Methods	Parameters
Proposed	population size = 10; ch_length = 2; xmin = zeros(population size,ch_len); xmax = ones(population size,ch_len); initsol = unifrnd(xmin,xmax); itermax = 25; NP = size(X,1);% population size ND = size(X,2);% number of decision variables netak = 0.5;% a value in the interval [0,1]; alphak = 0.5;% rate of momentum or inertia coefficient that has a value in the interval of [0,1] betak = 0.5;% velocity limiter ratio for stage k delT = 0.1; Leader_score = inf; V = 0.5; u = 0.0265; r0 = 10; r = r0 + u*to; omega = 0.005; phi0 = 3*pi/2; sig = [0.25 0.6 1];
AO	alpha = 0.1; delta = 0.1;
PRO	ub = U(1,:); lb = L(1,:); %maximum and minimum values of solution POP = val; nPOP = N/2; %%%size of main population rPOP = 0*ones(nPOP,D); %%% rice population pPOP = 0*ones(nPOP,D); %%% poor population gRc = 0;gPc = 0;bRc = 0;bPc = 0;
CSO	popSz = size(partMat,1); MR = 0.75; % Mixture ratio to decide tracking or seeking mode, % here it is the ratio of total seeking mode cats to total popSz CDC = 0.65; % Counts of dimensions to change SRD = 0.25; % Selected range of dimensions SMP = 5; c = 2.05; wMax = 0.9; wMin = 0.3; % particle matrix and percentage as specified perCnt = 0.25;
HGS	VC2 = 0.03; %The variable of variation control sumHungry = 0;%record the sum of each hungry
SSO	NP = size(X,1);% population size ND = size(X,2);% number of decision variables netak = 0.5;% a value in the interval [0,1]; alphak = 0.5;% rate of momentum or inertia coefficient that has a value in the interval of [0,1] betak = 0.5;% velocity limiter ratio for stage k delT = 0.1; Leader_score = inf; V = 0.5;

**Pseudo-code for AISSO:** 
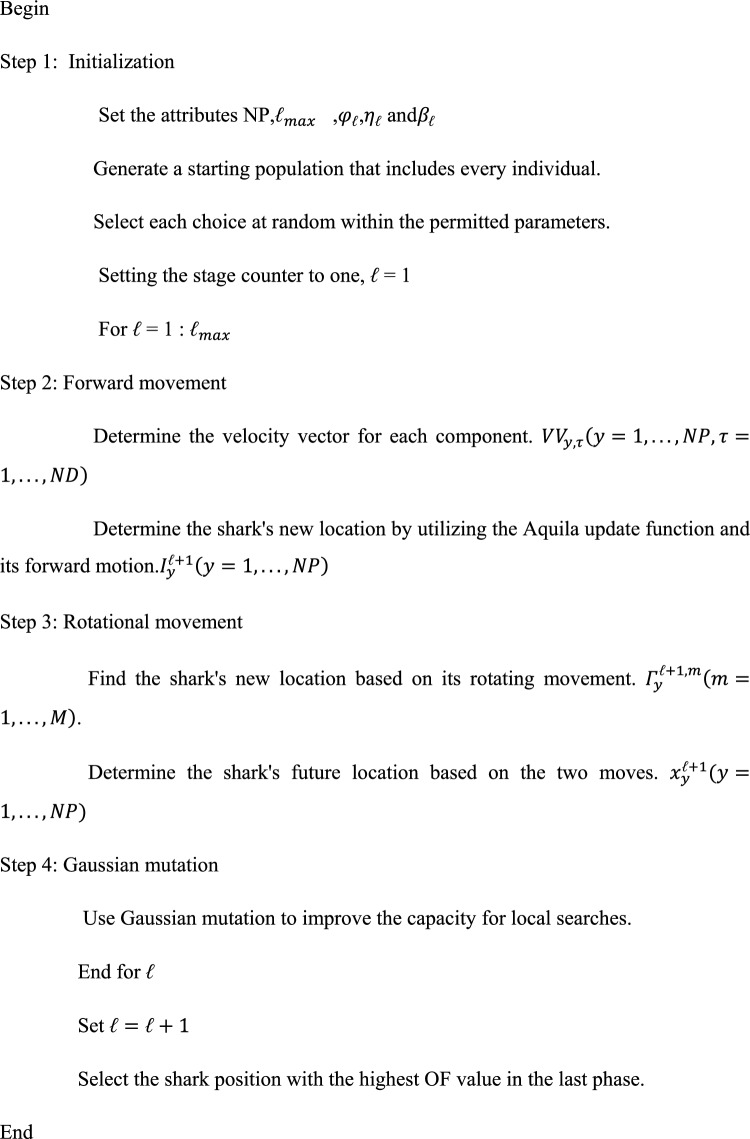


## Results and discussion

This section covers the results and discussion.

### Simulation setup

The MATLAB tool has been used to implement the proposed work. In this research, two datasets were employed. The AISSO-based PPI prediction algorithm we have presented has been analyzed and its performance matrices compared with traditional methods like Aquila^45^, Cat Swarm Optimization (CSO)^46^, Hunger Games Search (HGS)^47^,Poor Rich Optimization (PRO)^48^, and Shark Smell Optimization (SSO)^49^.

### Dataset description and pre-processing

We use the datasets of two species, Saccharomyces cerevisiae (SC) and Escherichia coli (EC), which each have a single dataset for the Biological Process (BP), Molecular Function (MF), and Cellular Component (CC) ontologies. Proteins lacking any GO annotations are removed. 10,831 and 6954 train proteins and 5436 and 1834 train proteins are present in SC and EC’s BP, MF, and CC databases, respectively. MetaGO Deducing Gene Ontology from multi-source pipelines are used^40^. The datasets include standard UniProt proteins annotated with experimental GO and predicted structural models using I-TASSER. Further, the resultant GO terms are analyzed using the LCA method to find the protein pairs with positive and negative GO terms for further PPI prediction.

### Convergence analysis

**Fig. 4:**
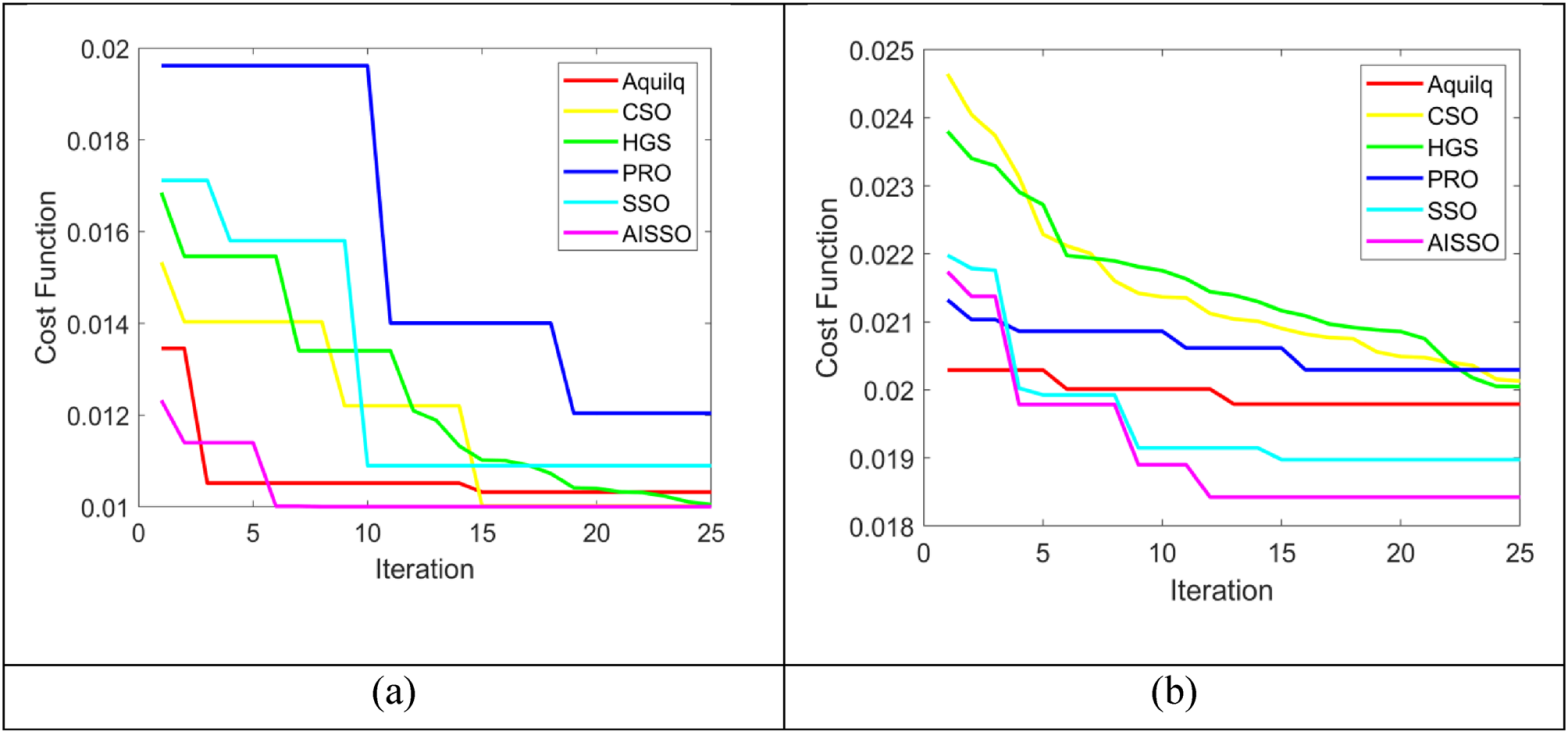
Cost Function comparison of the proposed AISSO strategy with conventional algorithms for (**a**) E. Coli and (**b**) S. Cerevisiae.

To assess the difference between the predicted as well as actual values, we assessed the cost functions of 0–25 iterations for two datasets, and the findings of the proposed AISSO PPI prediction are compared with optimizations such as Aquila, HGS, PRO, CSO, as well as SSO, as seen in Fig. 4. The E. Coli findings reveal that for iterations 0–5, the cost function values for PRO and HGS were high, ranging from 0.018 to 0.02, while our proposed AISSO strategy had a rate of 0.01 to 0.012. When the CSO and HGS methods provide cost function values ranges in 0.025–0.03, the proposed AISSO approach provides cost function values ranges in 0.01–0.015, demonstrating that the proposed AISSO Sequence-dependent PPI prediction method can provide more accurate results than other optimization techniques.

### Performance analysis

**Fig. 5:**
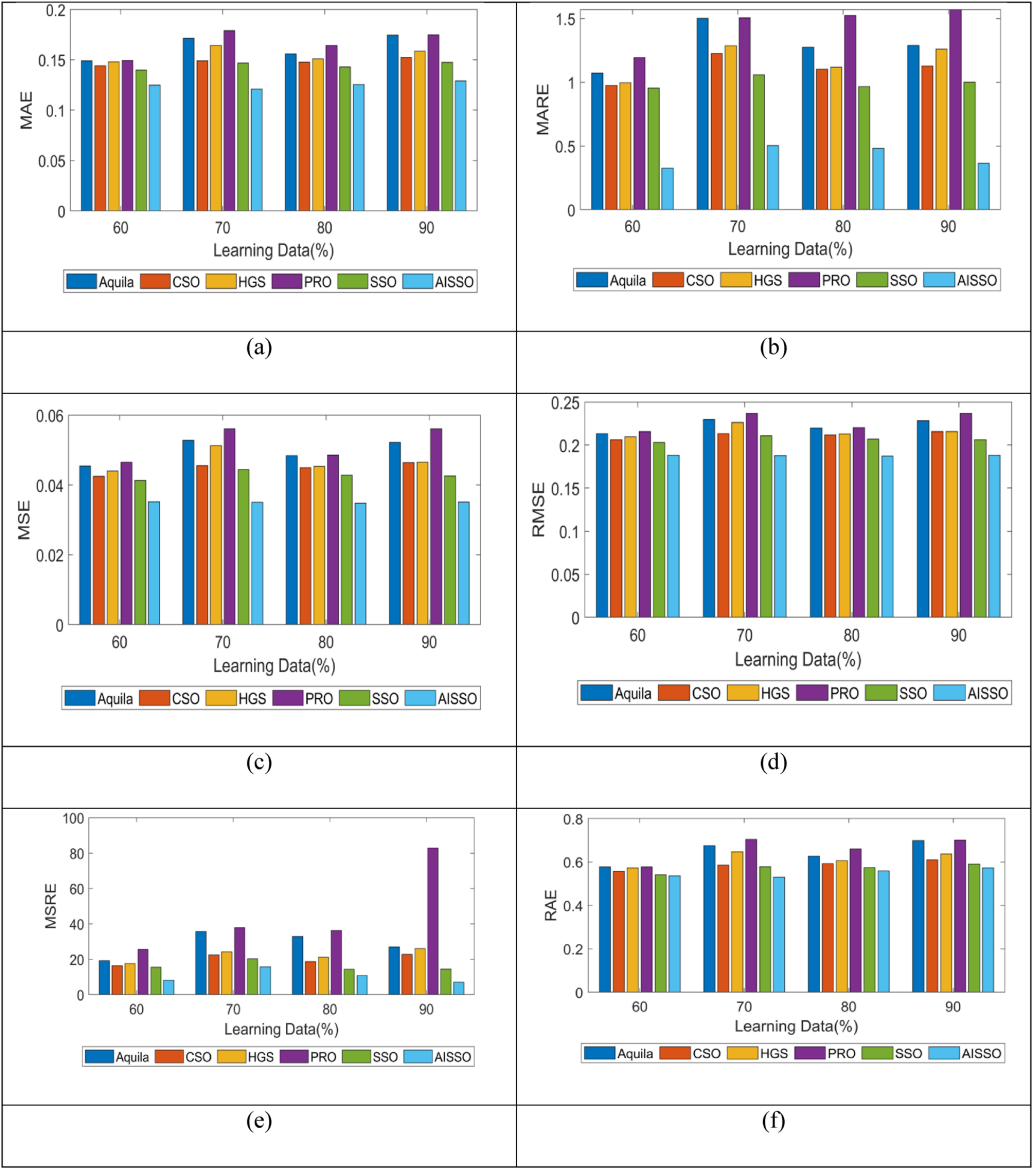
Comparison of our proposed AISSO-based PPI prediction model with standard optimization algorithms in terms of (**a**) MAE, (**b**) MARE, (**c**) MSE, as well as (**d**) RMSE (**e**) MSRE, (**f**) RAE for E. Coli.

The performance matrices MAE, MARE, MASE, MSRE, RAE, as well as RMSE of the proposed AISSO method, were contrasted with the conventional optimization techniques such as Aquila, CSO, HGS, PRO, and SSO for E. Coli, as shown in Fig. 5. When the PRO and Aquila MAE values are 0.017 and 0.015, respectively, our proposed AISSO-based prediction technique achieves 0.014 at 60 learning percentage (LP), which is lower than other traditional methods.

For 60 and 70 LPs, our proposed AISSO-based prediction technique yields a MARE value of 1, demonstrating the efficiency of the proposed AISSO PPI prediction strategy. The SSO approach produces MSE and RMSE values of 0.054 and 0.2 for 60LP, respectively, which are greater than our proposed AISSO-based approach, demonstrating that our AISSO-based method is more reliable and can deliver superior performance than methods.

**Fig. 6:**
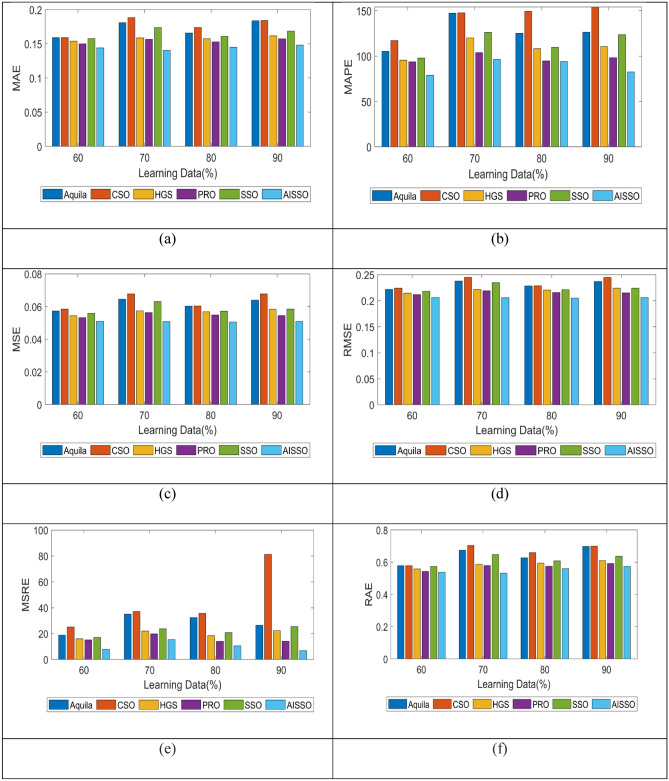
Comparison of the proposed AISSO-based PPI prediction approach with traditional optimization algorithms for S. Cerevisiae in terms of (**a**) MAE, (**b**) MARE, (**c**) MSE, (**d**) RMSE, (**e**) MSRE, (**f**) RAE.

Similarly, the MAE, MARE MASE, and RMSE values of our proposed AISSO-based approach were evaluated for S. Cerevisiae, and the results were compared with conventional optimization algorithms, shown in Fig. 6. When 60–90 LPs, the Aquila algorithm produces MAE values of 0.17, 0.18, 0.18, and 0.17, our proposed strategy obtains lower values of 0.17, 0.17, 0.15, and 0.16. MARE values for S. Cerevisiae are likewise lower for all LPs, 1.1, 1.1, 1, and 1.5, indicating that our developed AISSO technique can deliver excellent results for this PPI prediction task. When the SSO and HGS techniques get greater MAE and MARE values, our proposed AISSO approach produces lower values, demonstrating that our AISSO-based PPI prediction strategy outperforms other traditional methods.

### Analysis of different classifiers and ablation studies

For multiple cases, we compared the effectiveness of the proposed AISSO-based approach with E. Coli and S. Cerevisiae, and the results are shown in Tables 4 and 5. Without optimization, our AISSO-based prediction strategy produces MAE and RMSE values of 0.1385 and 0.1921, respectively, but with cosine similarity, the rates are 0.1684 and 0.2190. When LSTM, GRU, CNN, and SVM acquire high MARE values of 2.4469, 2.2261, 2.9994, and 1.9701, respectively, our proposed AISSO-based prediction strategy yields a lower value of 0.1539.

**Table 4:** Performance comparison of our proposed AISSO strategy with E. Coli for diverse scenarios.

Parameter	Proposed with cosine similarity	Proposed without optimization	LSTM	GRU	CNN	SVM	Proposed PPI prediction	GCN	DL-PPI
MAE	0.168	0.138	0.297	0.276	0.238	0.344	0.136	0.204	0.1932
RMSE	0.219	0.192	0.357	0.328	0.275	0.423	0.198	0.2545	0.2409
MARE	1.896	1.607	2.446	2.226	2.999	1.970	1.153	1.4191	1.3434
MSE	0.047	0.036	0.128	0.108	0.076	0.179	0.039	0.0863	0.0817

**Table 5:** Performance comparison of our proposed AISSO strategy with S. Cerevisiae for diverse scenarios.

Parameter	Proposed with cosine similarity	Proposed without optimization	LSTM	GRU	CNN	SVM	AISSO based	GCN	DL-PPI
MAE	0.166	0.182	0.272	0.429	0.237	0.341	0.175	0.185	0.256
RMSE	0.234	0.248	0.339	0.506	0.275	0.407	0.230	0.2307	0.322
MARE	1.396	1.861	1.892	1.258	2.926	2.783	1.732	1.2867	1.844
MSE	0.054	0.06	0.115	0.256	0.075	0.165	0.053	0.0783	0.102

Similarly, for S. Cerevisiae, performance indices including MAE, RMSE, MARE, and MSE values were examined for multiple situations, and the outcomes are shown in Table 4. With S. Cerevisiae, our proposed approach yields MAE and RMSE values of 0.17534 and 0.23042, whereas other networks such as LSTM, GRU, CNN, and SVM reach rates of 0.27209, 0.42922, 0.23714, and 0.34126 and 0.33928, 0.50627, 0.27504, and 0.44073, demonstrating that our novel approach surpasses other scenarios.

### Statistical analysis

Statistical analysis is typically used in a data set to assess and comprehend results and explain data fluctuations. In our work, we conducted statistical analyses including best, worst, mean, median as well as STD of several optimization algorithms, including Aquila, HGS, PRO, CSO, and SSO for E. Coli and S. Cerevisiae, and also the outcomes were compared with our proposed AISSO approach as seen in Table 6, 7. When Aquila and HGS optimizations yield best and worst values of (0.0102, 0.0102) as well as (0.0100, 0.0167), respectively, for E. Coli, our proposed AISSO based prediction technique obtains best and worst values of 0.0100 and 0.0106.

**Table 6:** Statistical analysis of our proposed AISSO-based strategy vs. other optimization algorithms for E. Coli.

Statistical analysis	Aquila	HGS	PRO	CSO	SSO	AISSO
Best	0.010222	0.010091	0.01019	0.010276	0.010084	0.010097
Worst	0.010222	0.016729	0.018511	0.019461	0.015773	0.010663
Mean	0.010222	0.011111	0.013114	0.012393	0.010934	0.010188
Median	0.010222	0.010342	0.012275	0.010276	0.010927	0.010097
STD	7.08E-18	0.002015	0.002613	0.003249	0.001282	0.000212

**Table 7:** Statistical comparison of our proposed AISSO-based strategy to extant optimization algorithms for S. Cerevisiae.

Statistical analysis	Aquila	HGS	PRO	CSO	SSO	AISSO
Best	0.010284	0.010106	0.010102	0.010485	0.010138	0.010012
Worst	0.015891	0.029087	0.026908	0.012794	0.019354	0.012694
Mean	0.010671	0.014784	0.013799	0.010762	0.010614	0.010425
Median	0.010284	0.013719	0.013134	0.010485	0.010167	0.010323
STD	1.11E-03	0.004046	0.003787	0.000766	0.001833	0.000697

Also, the mean and median values of our proposed AISSO-based prediction technique were 0.0101 and 0.0100, respectively, lower than those of the other optimisation methods’ mean and median values. Similarly, for S. Cerevisiae, our AISSO-based prediction strategy obtains statistical analysis values of 0.0100, 0.0126, 0.0104, 0.0103, and 0.0006, whereas traditional methods produce high values, demonstrating that our AISSO-based prediction approach outperforms existing optimization algorithms.

### Analysis of accuracy

**Fig. 7:**
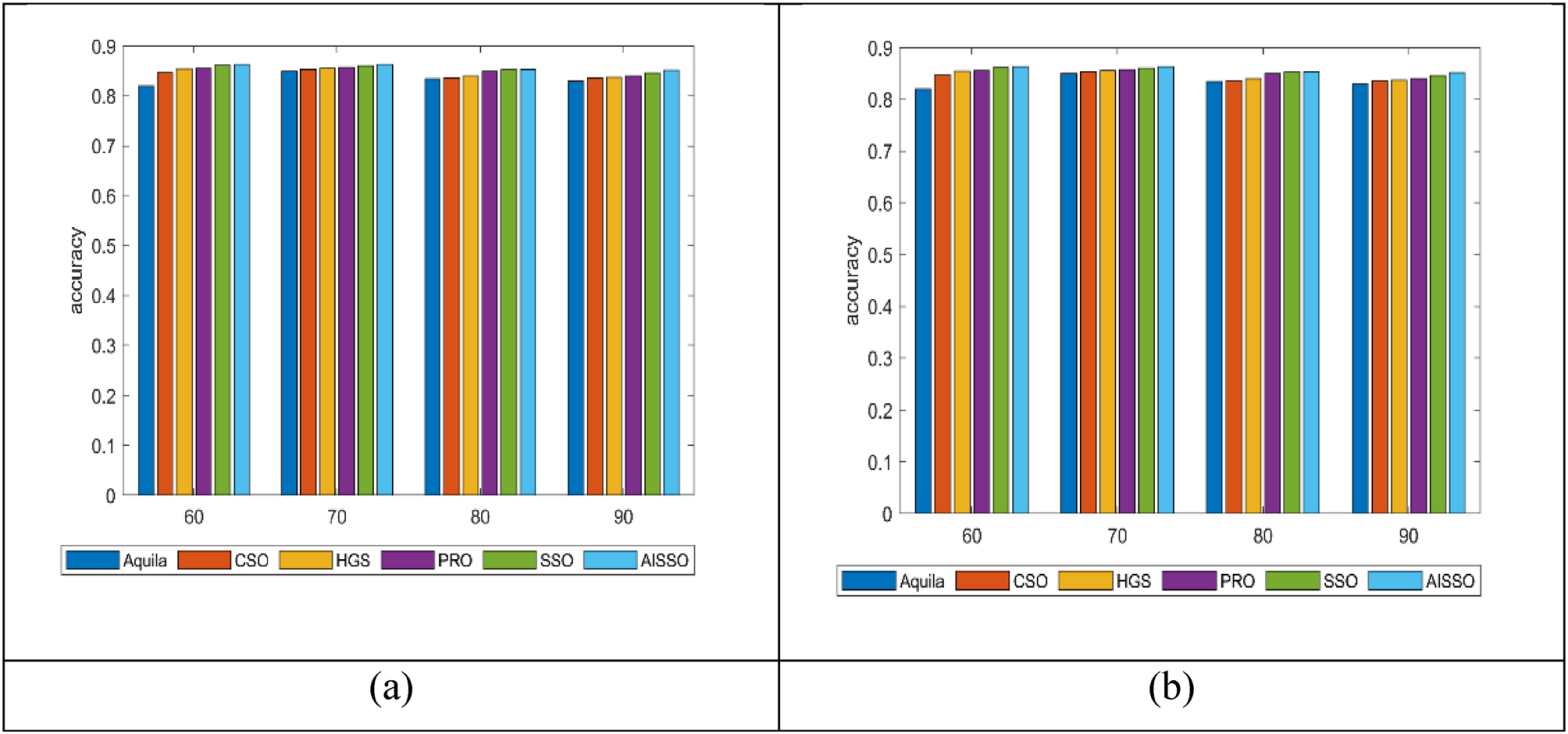
Comparison of the proposed AISSO-based PPI prediction approach with traditional optimization algorithms (**a**) E. Coli and (**b**) S. Cerevisiae.

The projected model’s performance is evaluated for E. Coli and S. Cerevisiae using different learning percentages, namely 60, 70, 80, and 90. According to the findings collected, the projected model has achieved the maximum accuracy for varying learning percentages compared to the conventional models. The obtained results are illustrated in Fig. 7. At 60 percent of the learning percentage, the developed model has attained enhanced accuracy (~ 87.37), which is much better than the conventional approaches such as Aquilla, CSO, HGS, PRO and SSO, respectively for E. Coli. Also, based on the accuracy of the developed model for S. Cerevisiae at LR 80, the developed model obtained the highest accuracy compared to the traditional methods.

### Local and global optima analysis

**Fig. 8:**
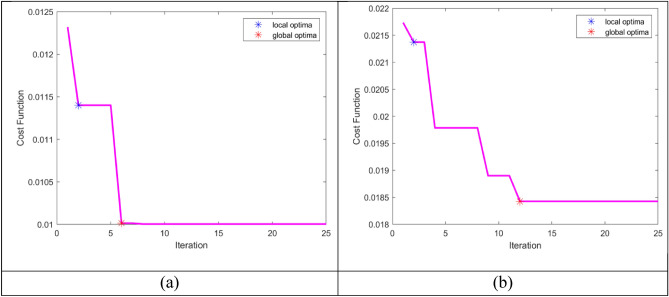
Analysis of Local and Global Optima.

An extrema (highest or minimum) point of the objective function for a specific area of the input space is known as a local optimum. The maximum or lowest value the objective function can accept throughout the whole input space is known as the global optimum. The global optimum is best for the system’s overall performance, whereas the local optimum is best for the performance of a single component. Figure 8 shows the local and global optima. Finding the minimum or maximum over the specified set, as opposed to local minima or maxima, is how global optimization differs from local optimization. Using traditional local optimization techniques, determining an arbitrary local minimum is quite simple.

### Computational time analysis

Table 8 shows the computational time analysis. For dataset 1, the proposed model shows the minimum computational time (~ 23.084) when compared to the other existing methods like AO (~ 73.78), CSO (~ 27.596), HGS (~ 64.284), PRO (~ 51.407) and SSO (~ 51.44). In addition, for dataset 2, the proposed model is 5%, 41%, 34%, 21%, and 5.5% better than the traditional methods like AO, CSO, HGS, PRO, and SSO. Thus, the proposed model is better than the other existing methods.

**Table 8:** Analysis of computational time.

Dataset 1	
AO	73.785
CSO	27.596
HGS	64.284
PRO	51.407
SSO	51.444
AISSO	23.084
Dataset 2	
Aquila	30.337
CSO	33.944
HGS	64.591
PRO	51.047
SSO	35.361
AISSO	29.785

## Conclusion

Protein–protein interaction prognostication is a subject that combines genomics with systems biology to detect and classify physical connections among protein groups or pairs. While numerous high-throughput experimental methods have been created to forecast PPIs, they have drawbacks, such as being expensive, time-consuming, and using incorrect and ineffective data and classifiers. Therefore, we introduce a novel PPI prediction methodology in this research that comprises two working phases: feature extraction and prediction. In the first step, we used the semantic similarity technique to provide new features that will help in accurate prediction, in addition to the conventional characteristics such as sequence-dependent and Gene ontology. AISSO neural networks like DBN and improved RNN were utilized in the second stage for better prediction.

Furthermore, this work created a unique optimization termed Aquila Influenced Shark Smell (AISSO) to deliver a better and more trustworthy prediction by optimising the weighting factors. The outcomes of the proposed PPI prediction strategy were contrasted with traditional methodologies, demonstrating that our novel method potentially delivers better results.

### Future scope

Additional pre-trained language models will be investigated in future work to produce protein sequence embeddings. We will also investigate the application of other protein information sources, like gene co-expression, which can be used as a node feature vector in a PPI network graph. Combine sequence data with other biological data types like structural details, gene expression profiles, or functional annotations for more thorough predictions. Improve the interpretability of your models to shed light on the underlying processes that underlie your forecasts. Examine how PPI prediction models are used in personalized medicine and medication development.

## Human subject

This study does not involve human subjects.

## Data Availability

The dataset is available with the corresponding author and available at individual request.
